# Extractable pool of biochar controls on crop productivity rather than greenhouse gas emission from a rice paddy under rice-wheat rotation

**DOI:** 10.1038/s41598-018-19331-z

**Published:** 2018-01-16

**Authors:** Punhoon Khan Korai, Xin Xia, Xiaoyu Liu, Rongjun Bian, Morris Oduor Omondi, Alphonse Nahayo, Genxing Pan

**Affiliations:** Center of Biomass and Biochar Green Technology, Institute of Resource, Ecosystem and Environment of Agriculture, Nanjing Agricultural University, 1 Weigang, Nanjing, 210095 China

## Abstract

The role of extractable pool of biochar in crop productivity and soil greenhouse gas (GHGs) emission is not yet clear. In this study, two biochars with and without extraction was added to a paddy before rice transplantation at 20 t·ha^−1^. Crop yield, plant traits and greenhouse gas emission monitored throughout a rice-wheat rotation. Between the biochar treatments, changes in bulk density and microbial biomass carbon were insignificant. However, the increase in organic carbon was similar between maize and wheat biochars while higher under bulk wheat biochar than extracted one. The increase in available P and K was higher under wheat than maize biochar regardless of extraction. Moreover, the increase in plant traits and grain yield, in rice season only, was higher under bulk than extracted biochars. Yet, there was no difference in changes in GHGs emission between bulk and extracted biochars regardless of feedstock. Nevertheless, increased methane emission for rice season was lower under extracted biochars than bulk ones. Overall, crop productivity rather than GHGs emission was affected by treatment of extraction of biochars. Thus, use of unextracted biochar is recommended for improving soil crop productivity in the paddy soils.

## Introduction

Globally, the agricultural soils and fragile soils with low resilience are at risk of degradation and ecosystem failure under climate change and crop production intensification^1,2^. Key soil constraints include low organic carbon storage, shortage of nutrient, and changes in ecosystem services, mainly in African, Middle Eastern, South American, and Asian countries^2^. Depletion of soil nutrients and reduction of soil organic carbon are common also across mainland China, central and southern Europe, Mid-East Africa, and Mid-South America as well as in the middle and western regions of the United States of America^3–5^. To act against this, biochar typically as a fine granular organic carbon mixture, is being tested as a soil amendment to improve soil fertility. Researchers have shown that such carbon can be stored in soil for hundreds of years^6,7^, helping improve soil fertility and provide many other benefits for crop production. A common view among proponents of biochar is that its use should be up-scaled to help reduce or stabilize atmospheric concentration of carbon dioxide.

The stable but porous biochar is known to be a soil conditioner for improving soil aggregation, porosity and buffering capacity thus helps improve soil fertility and micro-biological activity^8^. The small pool of water extractable carbon could increase carbon substrates for microbial metabolic use and thus potentially increase microbial respiration^9^. In addition, water extractable pool of biochar has been shown to contain plant growth promoting agents and thus could be used as a liquid fertilizer^10–12^. However, washing to remove water soluble components could modify biochar’s chemical composition and thus potentially the functions on nutrient availability to crops^13^. Therefore, understanding if an extractable pool would affect biochar’s role in improving soil fertility and soil gas emission as well as plant performance could be a research need of priority before commercialization of value-added biochar products for agriculture.

Rice (*Oryza sativa L*.) is an important and major food crop cultivated in the majority of Asian countries including China^14^. Use of biochar from crop straw has been shown to increase rice yield and N use efficiency while reducing GHGs emission^15^. However, there are cases of short term increase in methane emission^16^. Biochar’s effects on crop growth and yield, soil properties, metal immobilization and on GHGs emission reduction have widely been examined in Asian countries^17^. It is so far not clear if an extractable pool manipulates biochar’s functioning for crop growth, soil fertility and GHGs emission, thus limiting the scaling up of biochar use in rice agriculture in Asian countries.

Here, we hypothesize that: firstly, extraction of biochar with removal of some extractable OC fractions and water soluble nutrients would modify biochar’s role in supplementing organic/inorganic nutrients and thus in promoting crop growth; Secondly, such extraction could affect biochar’s role in improving soil OC stock and as well as in reducing soil respiration and GHGs such as methane emission from rice paddy; Thirdly, the extraction induced change in the above effects could last shortly as rice is cultivated under seasonally flooding condition and such effects would differ between different biochars containing different size and composition of a water extractable portion.

In this study, through a comparative study of a rice paddy across a whole cycle of rice-wheat rotation, we aimed to address if a water extractable pool of biochar affects soil physico-chemical properties, crop traits and yield, and soil respiration and greenhouse gas emissions and if these changes differ between types of biochar, and time span of the rice-wheat rotation system. This information would be of critical value for developing biochar products for enhancing crop productivity and climate stabilization in rice agriculture.

## Results

### Soil properties changes

The relative change in soil physicochemical properties of soil with biochar treatments are shown in Table 2. These changes with biochar were more or less consistent across the two crop seasons though a reduction in soil bulk density was higher in wheat season than in rice season. The relative pH increase was not significantly different between crop seasons, biochar types and extraction treatment. Increase in soil organic carbon (SOC) was not significantly different between two types of biochars, but was 21–23% higher under bulk wheat biochar treatment (WBB) compared to extracted wheat biochar (WBE) treatment in both crop seasons.

**Table 1:** Basic properties and composition of the studied biochar with and without extraction.

Treatment	pH (H_2_O)	TOC (g·kg^−1^)	DOC (mg·kg^−1^)	TN (g·kg^−1^)	Available P (g·kg^−1^)	Available K (g·kg^−1^)
MBB	9.6	404.3	231.7	10.4	2.63	4.37
MBE	8.5	350.0	209.8	9.1	1.51	2.58
WBB	10.0	465.3	745.3	11.4	5.66	1.68
WBE	9.1	399.1	698.7	9.5	3.04	0.99

TOC, total organic carbon; DOC, dissolved organic carbon; TN, total nitrogen; MBB, bulk maize biochar; MBE, extracted maize biochar; WBB, bulk wheat biochar; WBE,extracted wheat biochar.

**Table 2:** Relative changes over control in soil physical and chemical properties under amendment of biochar with and without extraction.

Treatment	pH (H_2_O)	SOC (g·kg^−1^)	BD (g·cm^−3^)	TN (g·kg^−1^)	AVP (mg·kg^−1^)	AVK (mg·kg^−1^)	MBC (g·kg^−1^)
**Rice season**							
MBB	0.52 ± 0.17 a A	15.8 ± 1.61 a A	−0.14 ± 0.02 a A	0.14 ± 0.12 a A	2.39 ± 0.61 a B	7.75 ± 3.37 a B	0.10 ± 0.03 a A
MBE	0.48 ± 0.11 a A	15.4 ± 1.38 a A	−0.19 ± 0.04 b A	−0.11 ± 0.14 a A	0.73 ± 0.89 b B	6.50 ± 1.71 a B	0.06 ± 0.03 a A
WBB	0.47 ± 0.04 a A	18.0 ± 0.40 a A	−0.09 ± 0.02 a B	−0.02 ± 0.29 a A	3.88 ± 0.45 a A	14.0 ± 0.96 a A	0.14 ± 0.03 a A
WBE	0.59 ± 0.12 a A	13.9 ± 0.53 b A	−0.09 ± 0.03 a B	0.10 ± 0.27 a A	2.08 ± 0.47 b A	12.5 ± 0.96 a A	0.08 ± 0.06 a A
**Wheat season**							
MBB	0.65 ± 0.16 a A	14.8 ± 1.64 a A	−0.16 ± 0.03 a A	0.19 ± 0.11 a A	1.97 ± 0.89 a B	5.75 ± 4.86 a B	0.13 ± 0.03 a A
MBE	0.47 ± 0.10 a A	14.3 ± 1.37 a A	−0.18 ± 0.08 a A	−0.07 ± 0.43 a A	1.05 ± 0.89 a B	5.25 ± 4.11 a B	0.06 ± 0.03 a A
WBB	0.69 ± 0.07 a A	16.8 ± 1.37 a A	−0.10 ± 0.04 a A	0.03 ± 0.29 a A	4.38 ± 0.66 a A	15.0 ± 2.38 a A	0.13 ± 0.03 a A
WBE	0.71 ± 0.10 a A	13.3 ± 0.24 b A	−0.13 ± 0.03 a A	0.14 ± 0.27 a A	2.38 ± 0.48 b A	12.7 ± 2.27 a A	0.09 ± 0.08 a A

MBB, bulk maize biochar; MBE, extracted maize biochar; WBB, bulk wheat biochar; WBE, extracted wheat biochar. Lowcase letters indicate difference between bulk and extracted biochar from a single feedstock, and capital letters indicate difference between maize and wheat biochar for a single extraction treatment, respectively.

On the other hand, differences in the relative change in soil nutrients existed between crop seasons and biochar types as well as between extraction treatments. Except bulk maize biochar (MBB) and the extracted maize biochar (MBE) in the wheat season, increases in available P were higher under unextracted biochar than under extracted one by up to 50%. The increase in available K was higher under wheat biochar than under maize biochar by over 100%, regardless of extraction and crop seasons. There was no difference in the relative change in microbial biomass carbon between biochar types, extraction treatment and crop seasons.

### Changes in plant traits

The plant height was significantly increased with WBB than with WBE by up to 71.2% in the rice season, while other parameters were not changed with extraction of a biochar in both crop seasons. While there was no difference between extracted biochars, the plant height was significantly higher under WBB than under MBB by 96.2% and 81.8% respectively in rice and wheat seasons (Table 3). The change in leaf area index ratio showed no significant differences between MBB and MBE in both crop seasons but higher under WBB than under WBE by 80.5% and 102.1% in rice and wheat seasons, respectively. In contrast, WBB exerted a significant increase in LAI by 123.1% and 150.2% over MBB in rice and wheat season, respectively, though no difference between WBE and MBE in both crop seasons.

**Table 3:** Relative change over control in plant traits and grain yield under amendment of biochar with and without extraction.

Treatment	Plant height (cm)	LAI ratio (m^−2^)	TGW (g)	Grain yield (kg·ha^−1^)
**Rice season**				
MBB	3.43 ± 1.0 a B	2.16 ± 0.5 a B	2.81 ± 0.83 a A	1973.9 ± 395.2 a A
MBE	3.41 ± 1.3 a A	1.74 ± 0.1 a A	1.22 ± 0.71 b A	1641.2 ± 354.6 a A
WBB	6.73 ± 1.1 a A	4.82 ± 1.0 a A	3.62 ± 0.51 a A	2487.7 ± 118.0 a A
WBE	3.93 ± 0.8 b A	2.67 ± 0.9 b A	2.18 ± 0.65 b A	1445.7 ± 145.8 b A
**Wheat season**				
MBB	8.03 ± 3.4 a B	1.89 ± 0.4 a B	2.80 ± 0.79 a B	758.9 ± 161.8 a A
MBE	5.50 ± 1.3 a B	1.45 ± 0.3 a A	2.39 ± 0.71 a A	512.9 ± 135.4 a A
WBB	14.6 ± 2.4 a A	4.73 ± 0.4 a A	3.97 ± 0.38 a A	961.9 ± 224.2 a A
WBE	10.0 ± 3.2 a A	2.34 ± 0.6 b A	2.83 ± 0.84 b A	554.8 ± 221.4 a A

TGW, thousand grain weight; LAI, leaf area index; MBB, bulk maize biochar; MBE, extracted maize biochar; WBB, bulk wheat biochar; WBE, extracted wheat biochar. Lowcase letters indicate difference between bulk and extracted biochar from a single feedstock, and capital letters indicate difference between maize and wheat biochar for a single extraction treatment, respectively.

The 1000-grain weight (TGW) was significantly increased by 61.5% in MBB compared to MBE in rice season, but no significant difference in the wheat season. On the other hand, the WBB showed a 66% and 40.2% increase in TGW over WBE in rice and wheat seasons, respectively. There was no change in TGW of rice between wheat and maize biochars both with and without extraction. In the wheat season, however, WBB showed a 41.7% increase in TGW over MBB though insignificant for extracted biochars. Finally, grain yield was significantly increased by 72% under WBB compared to WBE in the rice season while there were insignificant differences for other treatments in both crop seasons.

As shown in Table 4, the plant root morphology was significantly affected by biochar treatments only in the rice season. Firstly, the root surface area was significantly increased under MBB than MBE by up to 124% in the rice season, but no change between WBB and WBE. Also, there was no difference in root surface area between maize and wheat biochar treatment of rice plant. Mean root diameter was significantly increased by 48% under WBB compared to WBE in the rice season while no differences between MBB and MBE. The mean root diameter of rice was significantly higher by 181.8% under WBB than under MBB, and by 250% under WBE than under MBE.

**Table 4:** Relative change over control in root morphology under amendment of biochar with and without extraction.

Treatment	Surface area (cm^2^)	Diameter (mm)	Specific length (cm/m^3^)	Volume/plant (cm^3^)
**Rice season**				
MBB	99.4 ± 14.2 **a A**	0.11 ± 0.09 **a B**	564.2 ± 31.3 **a A**	1.88 ± 0.17 **a A**
MBE	44.2 ± 10.8 **b A**	0.06 ± 0.06 **a B**	391.4 ± 34.4 **b A**	0.69 ± 0.13 **b A**
WBB	107.6 ± 33.7 **a A**	0.31 ± 0.07 **a A**	605.0 ± 28.8 **a A**	2.20 ± 0.15 **a A**
WBE	91.8 ± 25.1 **b A**	0.21 ± 0.08 **b A**	460.7 ± 40.4 **b A**	0.92 ± 0.12 **b A**
**Wheat season**				
MBB	18.9 ± 1.5 **a A**	0.09 ± 0.07 **a A**	126.5 ± 74.0 **a A**	0.24 ± 0.20 **a A**
MBE	16.8 ± 3.4 **a A**	0.07 ± 0.03 **a A**	93.0 ± 29.5 **a A**	0.24 ± 0.18 **a A**
WBB	24.0 ± 11.7 **a A**	0.09 ± 0.03 **a A**	128.6 ± 63.1 **a A**	0.36 ± 0.14 **a A**
WBE	21.7 ± 10.1 **b A**	0.08 ± 0.04 **a A**	82.8 ± 21.1 **b A**	0.07 ± 0.13 **b A**

MBB, bulk maize biochar; MBE, extracted maize biochar; WBB, bulk wheat biochar; WBE, extracted wheat biochar. Lowcase letters indicate difference between bulk and extracted biochar from a single feedstock, and capital letters indicate difference between maize and wheat biochar for a single extraction treatment, respectively.

For specific length in the rice season, there were insignificant differences between maize and wheat biochars. However, MBB and WBB showed a 44.1% and 31.3% increase compared to respectively MBE and WBE. Similarly, the root volume/plant was increased by 172% under MBB and by 139% under WBB compared respectively to MBE and WBE in the rice season. Again, no difference found between maize and wheat biochars in volume/plant in the rice season

### Soil respiration and GHG emissions

The differences between the total seasonal emission of CH_4_, N_2_O, and CO_2_ in rice and wheat cropping systems are exhibited in Table 5. Total emission of N_2_O was significantly decreased under WBE than under WBB by up to 10.3% in the rice season but such a difference not observed for MBB and MBE. Yet, total N_2_O emission was significantly decreased by 82% under WBB than under MBB, and by 91% under WBE than under MBE, respectively in the rice season. For the wheat seasons, however, the change with different biochars in N_2_O emission was insignificant between extraction treatments. Overall, the non-CO_2_ GWP and GHGI were not significantly changed between the biochar treatments in both crop seasons.

**Table 5:** Total CO_2_, CH_4_, and N_2_O emissions, and total global warming potential (non^−^CO_2_ GWP; kg·ha^−1^) and greenhouse gas intensity GHGI (kg·t^−1^ of rice wheat produced) from the rice-wheat growing season after under amendment of biochar with and without extraction.

Treat ment	CH_4_−C	N_2_O−N	CO_2_−C	GWP	GHGI
**Rice season**					
MBB	23.78 ± 5.0 **aA**	0.07 ± 0.13 **aA**	121.08 ± 14.9 **aA**	48.13 ± 4.10 **aA**	9.72 ± 0.9 **aA**
MBE	16.57 ± 4.7 **aA**	0.04 ± 0.11 **aA**	101.78 ± 10.4 **aA**	25.47 ± 4.02**bA**	3.96 ± 0.86 **aA**
WBE	30.75 ± 6.0 **aA**	−0.39 ± 0.01a**B**	113.14 ± 14.7 **aA**	50.26 ± 6.54 **aA**	6.04 ± 0.68 **aA**
WBE	16.24 ± 8.4 **aA**	−0.43 ± 0.01**bB**	111.59 ± 12.5 **aA**	40.23 ± 5.27 **aA**	4.59 ± 0.36 **a A**
**Wheat season**					
MBB	0.15 ± 0.06 **aA**	−0.32 ± 0.06 **aA**	137.63 ± 13.3 **aA**	21.63 ± 4.36 **aA**	14.72 ± 3.89 **aA**
MBE	0.13 ± 0.03 **aA**	−0.35 ± 0.10 **aA**	70.86 ± 11.2 **aA**	17.12 ± 2.26 **aA**	25.44 ± 6.34 **aA**
WBB	0.11 ± 0.07 **aA**	−0.30 ± 0.16 **aA**	247.95 ± 28.1 **aA**	29.41 ± 8.58 **aA**	15.31 ± 5.77 **aA**
WBE	0.07 ± 0.03 **a A**	−0.33 ± 0.04 **a A**	77.91 ± 14.8 **aA**	27.97 ± 7.58** aA**	46.76 ± 7.32 **aA**

Non-CO_2_ GWP, global warming potential calculated of non-CO_2_ gas emissions; GHGI, greenhouse gas intensity, calculated with non-CO_2_ GWP on the basis of yield; MBB, bulk maize biochar; MBE, extracted maize biochar; WBB, bulk wheat biochar; WBE, extracted wheat biochar. Lowcase letters indicate difference between bulk and extracted biochar from a single feedstock, and capital letters indicate difference between maize and wheat biochar for a single extraction treatment, respectively.

The data for overall changes in non-CO_2_ GWP, crop yield and GHGI of a whole rice wheat rotation studied are given in Table 6. Hereby, the rice yield was significantly increased by up to 71.2% under WBB than under WBE in the rice season but insignificantly changed between MBB and MBE. The wheat yield, however, showed insignificant differences regardless of biochar types or extraction treatment. The total yield of rice and wheat in the whole rotation showed a 72% increase under WBB compared to WBE though unchanged between MBB and MBE. Moreover, it was increased under WBB by 39.1% compared to MBB but unchanged between WBE and MBE. Nevertheless, the total non-CO_2_ GWP and GHGI of the whole rotation cycle were not significantly changed between the treatments, regardless of bicohar type and extraction treatment.

**Table 6:** Annual global warming potential ((non-CO_2_ GWP; t C·ha^−1^·year^−1^), yield (t·ha^−1^) and greenhouse gas intensity (GHGI; t C·t^−1^ grain yield year^−1)^ of the rice-winter wheat cropping rotation system under amendment of biochar with and without extraction.

Treatment	Rice season			Winter wheat season			Rotation cycle		
	GWP	Yield	GHGI	GWP	Yield	GHGI	GWP	Yield	GHGI
MBB	48.13 ± 4.1 **aA**	1.97 ± 0.4**Aa**	9.72 ± 0.9 **aA**	21.63 ± 4.6 **aA**	0.51 ± 0.6 **aA**	14.72 ± 3.8 **aA**	69.76 ± 6.7 **aA**	2.48 ± 0.4 **aB**	24.4 ± 3.5 **aA**
MBE	25.47 ± 4.0**bA**	1.64 ± 0.8 **aA**	3.96 ± 0.8 **aA**	17.12 ± 2.2 **aA**	0.76 ± 0.4 **aA**	25.44 ± 6.3 **aA**	42.60 ± 4.6** aA**	2.40 ± 1.0 **aA**	29.4 ± 2.8 **aA**
WBB	50.26 ± 6.5 **aA**	2.49 ± 0.2 **aA**	6.04 ± 0.6 **aA**	29.41 ± 8.5 **aA**	0.96 ± 0.5 **aA**	15.31 ± 5.7 **aA**	79.68 ± 7.5 **aA**	3.45 ± 0.4 **aA**	21.4 ± 6.9 **aA**
WBE	40.23 ± 5.2 **aA**	1.45 ± 0.5**bA**	4.59 ± 0.3 **aA**	27.97 ± 7.5 **aA**	0.56 ± 0.2 **aA**	46.76 ± 7.3 **aA**	68.20 ± 5.9 **aA**	2.00 ± 0.4 **bA**	51.4 ± 8.3 **aA**

Non-CO_2_ GWP, Global warming potential, calculated of non-CO_2_ gas emissions; GHGI, greenhouse gas intensity, calculated with non-CO_2_ GWP on the basis of yield; MBB, bulk maize biochar; MBE, extracted maize biochar; WBB, bulk wheat biochar; WBE, extracted wheat biochar. Lowcase letters indicate difference between bulk and extracted biochar from a single feedstock, and capital letters indicate difference between maize and wheat biochar for a single extraction treatment, respectively.

## Discussions

### Soil and plant effects

Biochar had been shown multiple benefits to soil fertility and crop growth^22^. And many reports addressed biochar induced changes in soil fertility and crop productivity both in pot and filed experiments^23^. Nevertheless, there were no comparison studies between extracted and unextracted biochar so far. Biochar amendment exerted significant changes in most of soil fertility parameters tested here (Supplement Table 1; Table 2). Being comparable to the report by Alburquerque *et al*.^24^, soil nitrogen content was not significantly elevated with biochar treatments in the present study, despite the relatively high soil organic carbon contents, of the amended soils. Interestingly, the relative changes with biochar amendment over control (Table 2), were significantly different between extracted and unextracted biochar and between maize and wheat biochars only in phosphorus availability. In a recent meta-analysis of literature studies, soil available phosphorus was elevated on average by 64%^25^.

**Figure 1:**
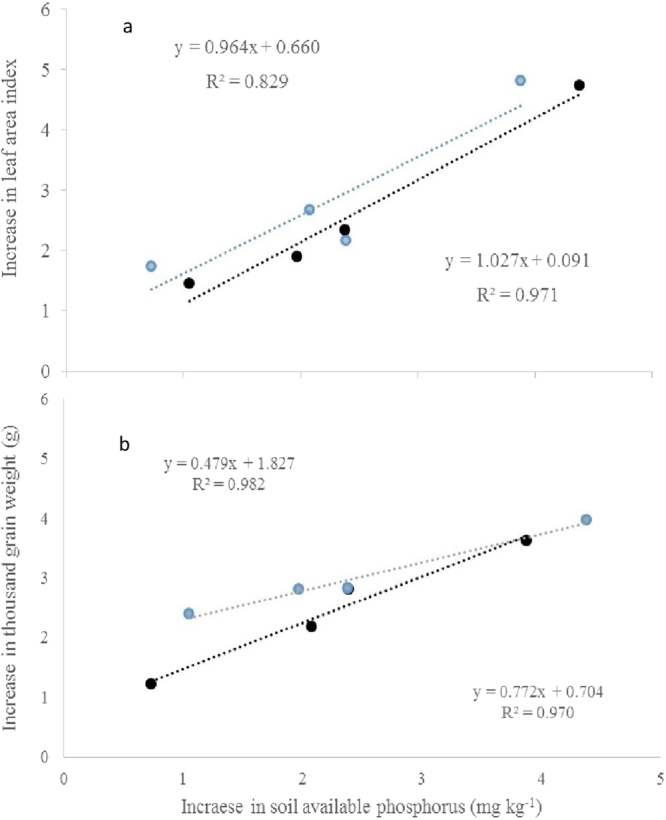
Correlation of increase in soil available phosphorus to increase in leaf area index (LAI) (**a**) and to increase in thousand grains weight (TGW) (**b**) of rice (blue circle) and wheat (black circle) with biochar amendment.

In the present study, soil available phosphorus contents were significantly higher under biochar treatment in both crop seasons, and higher under wheat biochar (WBB and WBE) than under maize biochar (MBB and MBE). Applied at 20 t·ha^−1^, both MBB and WBB increased markedly rice and wheat yield as compared to the relevant extracted biochar (Supplement Table 2; Table 3). Moreover, relative change only in thousand grain weight (TGW) was found to be significantly different both between biochars and between extraction treatments. Unfortunately, the grain yield increase was not shown significantly different between the treatments, being constrained by the high inter-plot variability. But the relative change in LAI and in TGW was found significantly correlated to that in available soil phosphorus pool, respectively in Fig. 1a and Fig. 1b. Therefore, the lower yield grain under extracted biochar amendment could be attributable to a decline in available phosphorus pool caused by extraction. In a field study with maize, soil available phosphorus was seen significantly increased under biochar blended fertilizer use^26^. Noting that soil available P pool could be enhanced in biochar amended soils^25^, the role of bicohar in soil fertility and crop productivity should be more explicitly explored though a lot of studies have emphasized on changes in soil organic carbon and N retention for crop productivity under biochar amendment^27^.

### SOC and GHG_S_

It had been highlighted that biochar soil amendment could be win-win strategy for enhancing organic carbon storage while reducing greenhouse gas emission particularly of N_2_O (nitrous oxide) in agricultural soil^1^. Nevertheless, the existence of a certain pool of labile carbon would impact on reduction of methane and N_2_O emission^28^. In our study, biochar amendment significantly enhanced the soil organic carbon measured for both rice and wheat seasons (Table 2; Supplement Table 1) by a degree proportional to the difference in biochar OC content across the treatments, depending on biochar types and extraction treatments. In detail, soil organic carbon enhancement was about 30% higher under bulk wheat biochar than under extracted wheat biochar in both rice and wheat seasons. In line with this, there was a significantly lower N_2_O emission and higher methane emission under extracted than under bulk wheat biochar in rice season. This could be explained by the findings that some labile organic carbon fractions could be utilized by methanogenic archaeal communities^28^, and contribute to prohibition for nitrifying bacterial communities as electron acceptors instead of nitrates^29^.

Unlike the finding of significantly increased soil respiration in the first year following a biochar application^30,31^, soil respiration measured as CO_2_ evolved was not changed with bicohar amendment in our study. This could be explained by, in part, that biochar used were conventionally ground into very fine particles and spread in dry croplands, resulting in increased soil respiration rate following the biochar accumulation^32^. In rice paddy with practice of hydroagric cultivation, biochar particles could be incorporated into soil aggregates under field conditions. In rice paddy field, organic matter can be physically protected in macro-aggregates and/or bound into soil minerals, becoming inaccessible to soil bacteria^33^. Accumulation of biochar under regular soil temperature and moisture conditions could also promote the development of soil microbes by making available exotic carbon sources^34^. In several cases, soil respiration was even adversely affected in bioenergy crop lands after biochar accumulation^35,36^. As the pool of carbon substrate available to microbes could be small in the biochar from completely charred crop straw, extraction treatment did not cause significant change in soil respiration when applied to the rice paddy soil at 20 t ha^−1^ before rice transplantation. The changes in annual crop yield between biochar treatments differed between rice and wheat seasons, though an increase of yield under bulk biochar treatments was seen to be consistent with the rice crop season but not with the wheat season. As an overall effect, however, greenhouse gas intensity of the whole cropping system was not different between bulk and extracted biochars treatments. Bulk maize and wheat biochars contained a small pool of organic molecules beneficial for crop growth^10,11^. Without a removal of extractable pool, both biochars here exerted a much greater beneficial effect on reducing non-CO_2_ GWP and GHGI while enhancing soil carbon storage, compared to extracted biochar for both rice and wheat crops. This again supported the accepted promising role of biochars^2,4^, generally without extraction, in mitigating climate change while sustaining crop productivity of rice-wheat cropping system^15^.

### Short term effect and crop seasons

The understanding of lasting effects of biochar on soil and ecosystem functioning was limited^37–39^. Meta-analysis studies had addressed significantly lower responses in subsequent years following a single amendment, in crop productivity^40^, soil respiration^41^, and metal immobilization^42^. And this had been attributed to the fast decline of the small pool of labile carbon existed in biochar^43,44^. For example, the increased methane emission from biochar amended rice soil disappeared in the second season following a single biochar application^16^. In this study, consistent seasonal differences in soil properties existed only of phosphorus available pool (Table 2; Supplement Table 1), which was greater in first rice season than in the subsequent wheat season. The changes in GHG emission were insignificant but consistent between the two seasons following a biochar application. That is, all these parameters were not shown time-dependent. The significant differences in root morphology (Table 4; Supplement Table 3) and some of plant traits (Table 3; Supplement Table 2) between bulk and extracted biochars were observed only in the first season of rice but not in the second crop season of wheat, over almost 12 months following the biochar amendment. Particularly of wheat biochars, higher increase in grain yield (Table 3) and lower GHG emission (Table 5; Supplement Table 4) was observed under bulk than under extracted biochar. The higher increases in root length, root volume and root surface under bulk than under extracted biochar could suggest a promotion of dissolved OC fractions as plant growth promoter^10–12^. As a small pool of labile OC would last very shortly^37–39^, the root promotion effect was not observed in the second crop here. Therefore, biochars without a removal of their labile OC fraction could exert greater benefits for crop productivity and soil nutrient retention especially of phosphorus though extraction did not cause a significant change in reduction of overall greenhouse gas emission. Otherwise, the labile OC could be extracted for foliar use and leave the extracted biochar to be used for soil metal immobilization^45^.

Furthermore, with regard to biochar types, this study suggest that wheat biochar has a larger portion of labile OC and available phosphorus pool, thus exerting a greater positive effect on soil nutrient, OC and plant growth as well as grain yield, compared to maize biochar. Recently, acid extraction from maize biochar (similar to that used in this study) showed a negative effect on plant seedling and root growth^12^.

This study demonstrated that extraction would affect plant growth and yield in the first crop without a tradeoff of soil OC storage and GHGs emission. Due to the high cost of a single biochar application at a dosage of 20 ton per hectare^46^, we could then recommend that biochar extraction for exotic use as valuable commercial product for foliar use for high value vegetables, medicinal plants as well as tea or fruit crops but leave the extracted biochar for soil reclamation, pollution control and soil cover for special use for green agriculture. By this way, biochar production and application could become more viable and provide an economic and eco-friendly solution for turning the bio-waste of crop straw into green.

## Conclusion

In this study, the effect of extraction on biochar performance in rice and wheat cropping system was determined. No significant changes in soil respiration but a universal enhancement in soil quality and crop performance in rice paddy soil was observed following a single amendment of maize and wheat with and without extraction respectively at 20 t·ha^−1^. Amendment of unextracted biochar enhanced soil fertility and crop productivity while unchanged greenhouse gas emission, compared to amendment of extracted biochar. This change supported the effect by dissolvable OC fraction and rich available phosphorus present in unextracted biochars. Thus the extractable pool of biochar pyrolyzed from crop residue could be considered a key player in soil fertility and crop productivity when addressing biochar’s role in agriculture. Future research should focus mainly on the interactions between variations in microbial community structure and carbon cycling in biochar amended soils with and without extraction treatment for valuable use of biochar in agriculture.

## Materials and Methods

### Biochar materials

Maize and wheat straw biochars were purchased from Sanli New Energy Company, Henan, China, where biochars were carbonized at pyrolyzing temperature of 350 °C–550 °C with a residence time of about 1 hour for the passage of the feedstock through the vertical kiln (5 m in height and 3 m diameter in diameter). Typically with this technology, 0.35 t of biochar, 250 kg of pyroligneous solution (wood vinegar) and 750 m^3^ of syngas, were obtained per metric ton of straw biomass. Biochar material was ground to pass a 2-mm sieve, homogenized and stored in a closed vessel under dry and cool condition before use for extraction.

### Biochar extraction

The biochar material from the stock was used for hot water extraction. In detail, a portion of biochar was mixed with tap water (1:20 w/v), heated to 80 °C and then shaken for 3 h. Subsequently, the aliquot was centrifuged and decanted. The resultant residue biochar was air-dried and stored at room temperature before analysis and applied under field conditions. As shown in Table 1, water extraction led to lower pH by almost 1 unit, a 12% reduction in TOC and total N, an over 40% reduction in available P and K though a 5–10% reduction in DOC, of both biochars.

### Field trial

A field experiment was conducted in a rice farm located at Kangbo village, Guli Township, Changshu-CS Municipality, Jiangsu, China (31°30ʹN, 120°33ʹE). The study area is located near the center of Tai Lake plain and characterized by subtropical monsoon weather with an average annual temperature of 16 °C and precipitation of 1100–1200 mm. The soil is a Gleyic Stagnic Anthrosol formed on clayey lacustrine deposit, which had been conventionally cultivated under rice-wheat rotation over centuries. The basic properties of the topsoil before experiment were as follows: soil pH (H_2_O), 7.3; soil organic carbon, 16.2 g·kg^−1^; total nitrogen, 2.1 g·kg^−1^; available phosphorous, 11.1 mg·kg^−1^; available potassium, 80.1 mg·kg^−1^; and bulk density, 1.2 g·cm^−3^.

A field trial was designed with amendment treatments of two straw biochars with and without water extraction respectively (MBB and MBE: bulk and extracted maize biochar; WBB and WBE: bulk and extracted wheat biochar) at 20 t·ha^−1^ compared to a control without biochar (CK). For soil amendment, both biochars were sprayed on the soil surface, mixed with soil by using a wooden rake, and then ploughed to a depth to 12 cm below surface, before rice transplantation. Each treatment plot was 3 × 6.5 m^2^ in area and in triplicates, arranged in a randomized complete block design. Each plot had separate irrigation inlets and drainage outlets. The field experiment was conducted across a whole cycle of rice^−^wheat production; farm management was following the local conventional performance, consistently across the treatments.

A rice cultivar Changyou 5 was sowed in all the plots in early June and harvested in late October of 2015. Following the rice harvest, a wheat cultivar of Yangmai 16 was sowed in early December 2015 and harvested in end of May 2016. Irrigation was not applied during the wheat growing season. For rice growth, N fertilizer as urea was applied at 337.5 kg N·ha^−1^, with two thirds as basal fertilizer before transplantation and another one third as top dressing. Phosphorus as calcium biphosphate and potassium as potassium chloride fertilizers were also applied as basal fertilizers at the rate of 375 kg P_2_O_5_·ha^−1^ and 375 kg K_2_O·ha^−1^, respectively. For wheat, fertilizers were applied as basal fertilizers at 187.5 kg N·ha^−1^ as urea, 375 kg P_2_O_5_·ha^−1^ as calcium biphosphate and 375 kg K_2_O·ha^−1^ as potassium chloride, respectively. However, a top dressing of urea at 225 kg N·ha^−1^ was additionally performed at the flowering stage during the wheat season.

### Crop traits and yield measurement

At ripening of each crop, 15 plants were randomly collected from a plot. Meanwhile, plant height and leaf area index of all the collected plants were measured instantly. Then the plants were separated into root, shoot and grain samples, all washed with distilled water and then and stored at 4 °C for further analysis. The root samples were used for measuring root length, surface area, average diameter and root volume, with an Instrument of Epson Expression 1640XL (Japan) equipped with Epson Twain Pro2.10 analysis software. Finally, all the plant samples were oven-dried at 105 °C for 30 min and further dried at 60 °C for 48 h^18^.

### Gas sampling and measurement

Soil respiration and greenhouse gas emission was monitored using a static chamber method^19^ across the crop growth periods of both rice and wheat. An aluminum flux collar was installed in each plot at a depth of 5 cm and the top edge was filled with water. When measuring, a chamber with height of 1 m covered the collar. The plastic chamber was covered with a layer of sponge and further covered with an aluminum foil to minimize potential heating inside the chamber during measurement. There was an electric fan to mix the air inside the chamber before gas sampling. A gas sampling was done between 8 to 11 am in a day throughout the crop growing season^19^. After a chamber closure and a pre-mixture of gases inside, a gas sample was collected after 0, 10, 20, and 30 min, respectively. The concentration of a given gas was analyzed using a modified gas chromatograph (Agilent 7980 A) equipped with flame ionization detector and an electron capture detector, allowing simultaneous determination of CO_2_, CH_4_, and N_2_O fluxes^15^. The slope of the mixing ratio changes in these four samples were used for gas flux calculation. Unless the samples yielded a linear regression value of greater than 0.90, sample sets were rejected. A flux between every two consecutive samplings was taken as the mean of the daily flux estimation for the period between the two intervals of all the monitored fluxes. The detailed calculation of a flux using these sequential samples was described by Zou *et al*.^19^.

Gas emission monitoring was operated in accordance with the water regime dynamics across growth stages. In detail, a gas sampling was done at seedling, tillering, stem elongation, heading, flowering, grain filling, and mature stage throughout the rice crop growing season and at the green-turning, stem elongation, heading, grain filling, and mature stages of the wheat crop growing season, respectively. All the single values of gas flux across the whole growing season was accumulated as a seasonal total emission as described by Zhang *et al*.^15^.

### Soil sampling and analysis

A composite topsoil sample was taken from each plot before sowing and at harvest both for rice and wheat growing season, respectively. The samples were sealed in polythene bags and shipped to laboratory within a day after sampling. After air-drying, the samples were sieved through 0.15-mm sieve. The pH, organic carbon, total nitrogen, available phosphorus, and available potassium of the soil samples were measured following the methods described by Lu^18^. Soil microbial biomass carbon was determined following a chloroform fumigation-extraction protocol, with a k_EC_ (the portion of microbial biomass carbon extracted by 0.5 mol·L^−1^ K_2_SO_4_ solution) of 0.45^20^.

### Calculation and statistics

Hereby, the global warming potential (GWP) was calculated using the following equation:

1 $${\rm{GWP}}({{\rm{kgCO}}}_{{\rm{2}}}/\mathrm{ha})=28\times {\rm{E}}{{\rm{CH}}}_{{\rm{4}}}=+265\times {\rm{E}}{{\rm{N}}}_{{\rm{2}}}{\rm{O}}$$

Where, Ech_4_ and En_2_O are, respectively, the total seasonal emissions of CH_4_ and N_2_O (kg ha^−1^), monitored over a rice and wheat growing seasons. The global warming potential of CH_4_ and N_2_O are 34 and 298 times greater than that of CO_2_ over a 100-year horizon^21^.

The greenhouse gases emission intensity (GHGI) was calculated using the equation:

2 $${\rm{GHGI}}({{\rm{kgCO}}}_{2}-{\rm{e}}/{\rm{t}})=\mathrm{GWP}/{\rm{Y}}$$

where GWP is the sum of CH_4_ and N_2_O (kg CO_2_–e/ha) emissions, and Y is the grain yield (t/ha).

The data are presented relative to changes in control and given as means ± SD. The difference between bulk and extracted biochar treatments and different biochars was tested using one-way analysis of variance. Level of significant difference was set at *P* < 0.05. Statistical analysis was performed using the IBM Statistical Package for Social Scientists (SPSS 20.0).
